# Comparative Methodological Evaluation of Three Bacteriophage Isolation Methods From Environmental Water Samples

**DOI:** 10.7759/cureus.108149

**Published:** 2026-05-02

**Authors:** Mohammod Johirul Islam, Mohammad Kamruzzaman, Fahim Alam Nobel, Md Fakhrul Islam Fahim, Saima Sabrina, Iftekhar Bin Naser

**Affiliations:** 1 Department of Biochemistry and Molecular Biology, Mawlana Bhashani Science and Technology University, Tangail, BGD; 2 Department of Biotechnology, School of Life Sciences, BRAC University, Dhaka, BGD

**Keywords:** bacteriophage, direct method, enrichment method, etec, klebsiella pneumoniae, peg method, pseudomonas aeruginosa, salmonella typhimurium, shigella sonnei, vibrio cholerae o1

## Abstract

Background: Bacteriophages are ubiquitous viruses that specifically infect and lyse bacterial hosts and have gained increasing attention for applications in biocontrol, phage therapy, food safety, and diagnostics. Efficient isolation of bacteriophages from environmental sources is essential for both research and applied purposes. This study aimed to comparatively evaluate the efficiency of three bacteriophage isolation methods from environmental water samples.

Methods: In this comparative methodological study, 96 environmental water samples were collected from four aquatic sites in Tangail, Bangladesh, over a one-year period (January-December 2025), with sampling conducted twice monthly. Six bacteriophages targeting clinically relevant bacterial hosts were assessed using three isolation methods: direct recovery, polyethylene glycol (PEG)-mediated precipitation, and host-based enrichment. Phage recovery efficiency was compared using Chi-square and Fisher’s exact tests, and effect sizes were estimated using odds ratios (ORs) with 95% confidence intervals (CIs).

Results: The average phage recovery rates were 9.38%, 21.35%, and 47.20% for the direct, PEG, and enrichment methods, respectively. A significant difference in recovery proportions was observed among methods (χ² = 224.1, df = 2, p < 0.0001). Pairwise comparisons demonstrated that the enrichment method yielded significantly higher recovery than the PEG method (OR = 3.29, 95% CI: 2.55-4.26, p < 0.0001) and the direct method (OR = 8.65, 95% CI: 6.28-11.97, p < 0.0001).

Conclusions: The modified enrichment method significantly improves bacteriophage recovery compared to the direct and PEG-based approaches, particularly for low-abundance phages. This method represents a robust and sensitive strategy for isolating bacteriophages from environmental water samples and may support future applications in phage-based therapeutics, environmental surveillance, and microbial control.

## Introduction

Bacteriophages are viruses that specifically infect and lyse bacterial hosts and are the most abundant biological entities in natural ecosystems [1]. Owing to their high host specificity and self-replicating nature, phages have attracted increasing attention for diverse applications, including biocontrol, phage therapy, bio-preservation, and diagnostics. In agriculture, food safety, aquaculture, and environmental management, phages are widely utilized as biocontrol agents to selectively target pathogenic bacteria [2]. Notably, bacteriophages have been approved for use as bio-preservatives in the food industry, exemplified by their application against Listeria monocytogenes in ready-to-eat meat and poultry products [3]. Their rapid replication and specificity further support their effectiveness in controlling foodborne pathogens across various matrices, including dairy, seafood, and water systems [4,5]. In the context of rising antimicrobial resistance, phage therapy has emerged as a promising alternative to conventional antibiotics [6]. In addition, phage-based platforms are increasingly being developed as sensitive and rapid diagnostic tools for detecting bacterial and viral pathogens [7,8]. Furthermore, phage-mediated control strategies may serve as preventive measures to limit the spread of infectious diseases and reduce environmental pathogen loads [9,10].

Natural aquatic environments, particularly sewage systems and riverine ecosystems, represent major reservoirs of bacteriophages targeting waterborne pathogens [11,12]. Efficient isolation of these phages is therefore essential for both fundamental research and applied applications. Importantly, systematic and quantitative comparisons of these isolation strategies under identical environmental conditions remain limited, particularly for clinically relevant host-phage systems. To our knowledge, no prior study has quantitatively compared these three methods under identical environmental conditions for these specific host-phage systems.

In this study, we comparatively evaluated three established bacteriophage isolation methods - direct recovery, polyethylene glycol (PEG)-mediated precipitation, and host-based enrichment - following targeted methodological refinements to improve recovery efficiency. A total of 96 environmental water samples were collected from four distinct aquatic sites in Tangail, Bangladesh, over a one-year period (January-December 2025). Six bacteriophages targeting clinically relevant bacterial hosts were isolated. The primary outcome was defined as phage recovery frequency across methods. We hypothesized that the enrichment method would yield higher phage recovery compared to the direct and PEG-based approaches. To our knowledge, this represents one of the first quantitative comparisons of these isolation strategies under standardized environmental conditions. The results demonstrate that the modified enrichment approach markedly improves phage recovery compared to the direct and PEG-based methods, supporting its utility as a sensitive and reliable strategy for isolating environmental bacteriophages.

## Materials and methods

Study design

This was a comparative methodological study designed to evaluate the efficiency of three bacteriophage isolation protocols (direct recovery, PEG-mediated precipitation, and host-based enrichment) using environmental water samples. The primary outcome was defined as phage recovery frequency, determined by successful plaque formation in drop spot assays.

Bacterial strains

Representative strains of enterotoxigenic *Escherichia coli* (ETEC), *Vibrio cholerae* O1, *Shigella sonnei*, *Salmonella typhimurium*, *Klebsiella pneumoniae*, and *Pseudomonas aeruginosa* were used as host bacteria for phage isolation.

Preparation of bacterial cultures

A single colony of each bacterial strain was inoculated into 5 mL of nutrient broth (NB) (HiMedia Laboratories, Mumbai, India) and incubated at 37 °C with shaking at 120 rpm for 16 hours to obtain overnight cultures. For log-phase cultures, 50 µL of the overnight culture was transferred into 5 mL of fresh NB and incubated at 37 °C with shaking at 120 rpm for four hours. These cultures were used for bacterial lawn preparation in phage detection assays [12-15].

Collection of environmental water samples

Environmental water samples were collected from four aquatic sites in Tangail district, Bangladesh: Tangail Medical College Sewage Line (TMCSL), Louhajang River, Pungli River, and Jamuna River. TMCSL represented a sewage-associated site, whereas the remaining three sites represented riverine environments. A total of 500 mL of water was aseptically collected from each site twice monthly from January to December 2025, yielding 96 environmental samples overall. Only freshly collected samples with a minimum volume of 500 mL were included in the study. Samples were transported immediately to the laboratory and processed on the day of collection. No samples were excluded from analysis.

Sample preparation with the direct method

From each of 500 mL of collected water samples, 10 mL was taken individually into a 15 mL centrifuge tube (Corning Incorporated, Corning, NY, USA) and centrifuged at 3000 rpm at room temperature (RT) for five minutes to precipitate the debris. The supernatant was then filtered with 0.22 µm membrane filters (Labfil, Hangzhou, Zhejiang, China) to remove the bacteria. The filtrate was then ultracentrifuged at 27000 rpm at RT for one hour with Sorvall Lynx 6000 (Thermo Fisher Scientific, Waltham, MA, USA) and followed by decanting all the filtrate slowly to keep the pellet untouched. The pellet was then resuspended with 500 µL of SM buffer (100 mM NaCl, 10 mM MgSO_4_, 50 mM Tris-HCl {pH 7.5}, and 0.01% {W/V} gelatin). The pellet was ready for the phage drop spot assay for the detection of bacteriophages. This protocol was adapted with minor modifications from previously described methods to improve phage concentration efficiency [12,15].

Sample preparation with the PEG-mediated precipitation

10 mL of each environmental water sample was centrifuged at 3000 rpm for five minutes at room temperature, and the supernatant was filtered through a 0.22 µm membrane filter. One-fourth volume of polyethylene glycol 6000 (PEG 6000) (Merck KGaA, Darmstadt, Hesse, Germany) was added to the filtrate, mixed thoroughly, and kept at 4 °C overnight. The mixture was subsequently ultracentrifuged at 27,000 rpm for one hour at room temperature. Following supernatant removal, the pellet was resuspended in 500 µL SM buffer for phage detection. This protocol was modified from previously published PEG precipitation methods [15] to improve phage recovery efficiency.

Sample preparation with the enrichment method

Similarly, 10 mL of each environmental water sample was centrifuged at 3000 rpm for five minutes at room temperature, followed by filtration through a 0.22 µm membrane filter. To the filtrate, 10 mL of 2× nutrient broth and 100 µL each of log-phase cultures of the six host bacterial strains were added. The enrichment mixture was incubated at 37 °C with shaking at 150 rpm for six hours to allow host-driven amplification of bacteriophages. Following incubation, cultures were centrifuged at 10,000 rpm for 10 minutes to remove bacterial cells, and the supernatant was re-filtered through a 0.22 µm membrane filter. The filtrate was ultracentrifuged at 27,000 rpm for one hour at room temperature. Pellets were resuspended in 500 µL SM buffer and used for phage detection. This enrichment protocol was adapted with methodological refinements from previously described procedures [16].

Preparation of bacterial lawn

Bacterial lawns were prepared using the double-layer agar method. Briefly, 500 µL of log-phase bacterial culture was mixed with 3.5 mL molten soft agar (0.8%) and immediately overlaid onto nutrient agar plates. Plates were allowed to solidify at room temperature for one hour before use in phage detection assays [12,15].

Phage detection by drop spot assay

For phage detection, 50 µL of each processed sample suspension was spotted onto the corresponding bacterial lawn and allowed to dry at room temperature for 30 minutes. Plates were incubated at 37 °C for 16 hours. The presence of bacteriophages was confirmed by the appearance of clear lytic zones (plaques) on bacterial lawns, indicating successful infection and lysis of the corresponding host strain. Personnel performing plaque assessment were not blinded to the isolation method used, which is acknowledged as a potential source of detection bias.

Statistical analysis

Bacteriophage recovery was expressed as counts and percentages of phage-positive observations for each isolation method. Differences in recovery proportions among methods were initially evaluated using the Chi-square (χ²) test based on aggregated frequency data. Pairwise comparisons between methods were performed using 2×2 contingency table analysis. Given the distribution of the data, a two-sided Fisher’s exact test was used to assess statistical significance. The magnitude of association between methods was quantified using odds ratios (ORs) with corresponding 95% confidence intervals (CIs). The cutoff p-value of <0.05 was considered statistically significant. All statistical analyses were performed using GraphPad Prism 8.0.1 (GraphPad Software, Inc., San Diego, CA, USA).

## Results

**Figure 1:**
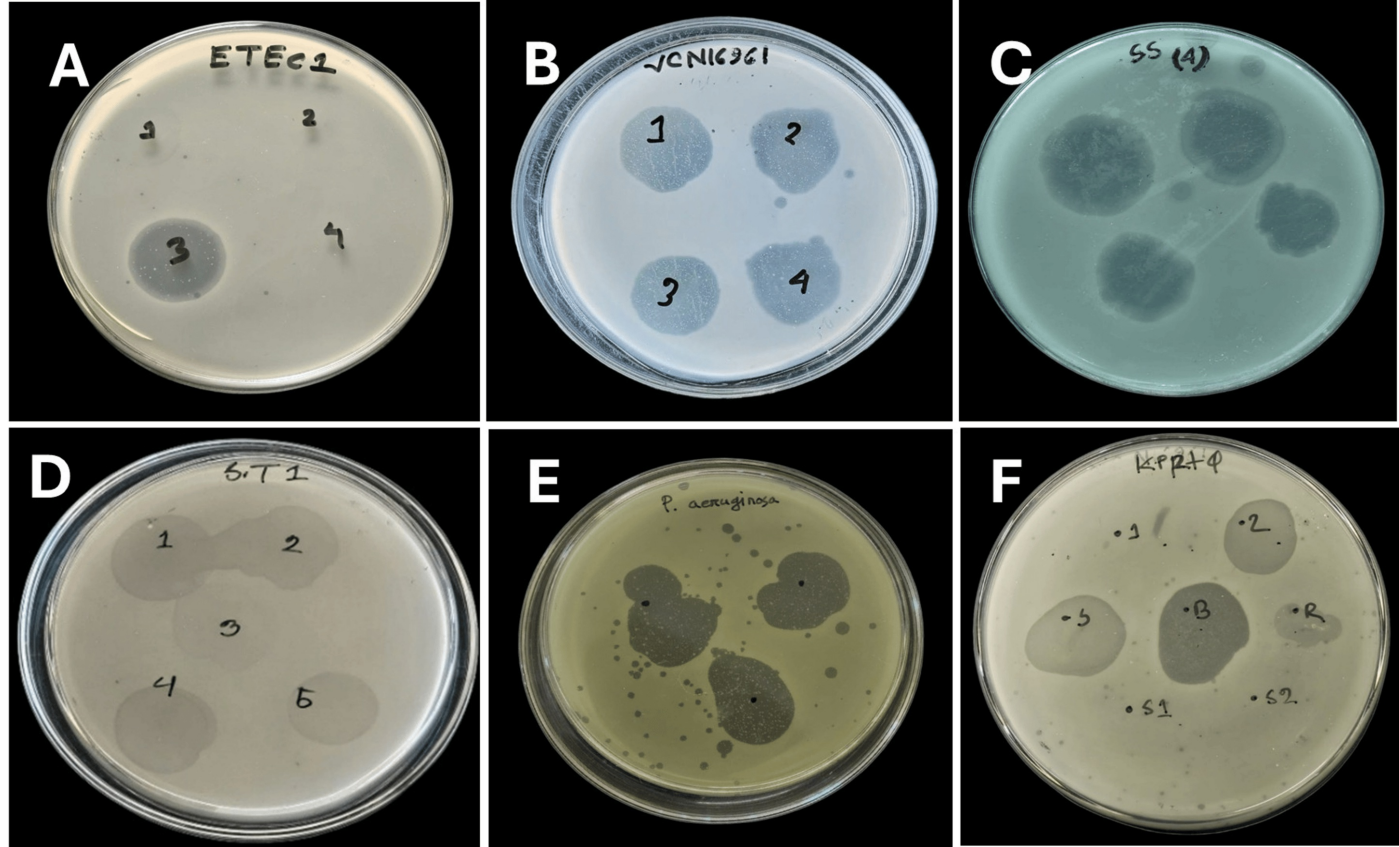
Representative figure for zones of lyses by different bacteriophages obtained from environmental water samples via three different customized methods on the lawns of respective bacterial cultures (A) ETEC ϕ, (B) VCO1 ϕ, (C) SS ϕ, (D) ST ϕ, (E) KP ϕ, and (F) PA ϕ. ϕ, bacteriophages; ETEC ϕ, enterotoxigenic *Escherichia coli*; VCO1 ϕ, *Vibrio cholerae *O1; SS ϕ, *Shigella sonnei*; ST ϕ, *Salmonella typhimurium*; KP ϕ, *Klebsiella pneumoniae*; PA ϕ, *Pseudomonas aeruginosa*.

Six types of bacteriophages (ϕ), namely enterotoxigenic *Escherichia coli* (ETEC ϕ), *Vibrio cholerae* O1 (VCO1 ϕ), *Shigella sonnei* (SS ϕ), *Salmonella typhimurium* (ST ϕ), *Klebsiella pneumoniae* (KP ϕ), and *Pseudomonas aeruginosa* (PA ϕ), were isolated from environmental water samples collected from four sites in Tangail district, Bangladesh, between January and December 2025. Phage detection was performed using host-specific bacterial lawns following processing of samples by three modified isolation methods (direct, PEG-mediated precipitation, and enrichment), as described in the "Materials and Methods" section. The presence of bacteriophages was confirmed by plaque formation in drop spot assays, observed as clear zones of lysis on bacterial lawns corresponding to each host strain (Figure 1).

A total of 96 environmental water samples were analyzed, corresponding to 576 sample-phage observations. Overall, phage recovery varied markedly depending on the isolation method and sampling site (Table 1). Across all samples, the enrichment method yielded the highest recovery rate (47.20%), followed by the PEG method (21.35%) and the direct method (9.38%). Statistical analysis demonstrated a significant difference in recovery proportions among methods (χ² = 224.1, df = 2, p < 0.0001). Pairwise comparisons confirmed that the enrichment method achieved significantly higher recovery than both the PEG method (odds ratio {OR} = 3.29, 95% CI: 2.55-4.26, p < 0.0001) and the direct method (OR = 8.65, 95% CI: 6.28-11.97, p < 0.0001).

**Table 1:** Frequency (%) and counts of bacteriophage recovery from environmental water samples using three isolation methods. Environmental water samples (n = 96) were collected from four sampling sites (24 samples per site) and screened for the presence of six bacteriophages using the direct, polyethylene glycol (PEG)-mediated precipitation and enrichment methods. Values represent the number of positive samples (counts) with corresponding percentages in parentheses, calculated relative to the total number of samples (n = 96) for each bacteriophage. The “Total (%)” for each method represents the overall recovery efficiency, calculated as the proportion of total phage detections across all phage–sample combinations (n = 576). PEG, polyethylene glycol; TMCSL, Tangail Medical College Sewage Line (TMCSL); ϕ, bacteriophages; ETEC ϕ, enterotoxigenic *Escherichia coli*; VCO1 ϕ, *Vibrio cholerae *O1; SS ϕ, *Shigella sonnei*; ST ϕ, *Salmonella typhimurium*; KP ϕ, *Klebsiella pneumoniae*; PA ϕ, *Pseudomonas aeruginosa*.

Methods	Sampling sites	Frequency of bacteriophage acquisition					
		ETEC ϕ	VCO1 ϕ	SS ϕ	ST ϕ	KP ϕ	PA ϕ
Direct method	TMCSL	6 (25.00%)	3 (12.50%)	2 (8.33%)	5 (20.83%)	6 (25.00%)	4 (16.67%)
	Louhajang River	5 (20.83%)	2 (8.33%)	1 (4.17%)	2 (8.33%)	2 (8.33%)	2 (8.33%)
	Pungli River	4 (16.67%)	1 (4.17%)	0 (0.00%)	0 (0.00%)	1 (4.17%)	2 (8.33%)
	Jamuna River	3 (12.50%)	1 (4.17%)	0 (0.00%)	0 (0.00%)	1 (4.17%)	1 (4.17%)
	Total (9.38%)	18 (18.75%)	7 (7.30%)	3 (3.13%)	7 (7.30%)	10 (10.42%)	9 (9.40%)
PEG method	TMCSL	9 (37.50%)	6 (25.00%)	4 (16.67%)	9 (37.50%)	11 (45.83%)	7 (29.17%)
	Louhajang River	8 (33.33%)	5 (20.83%)	3 (12.50%)	5 (20.83%)	6 (25.00%)	7 (29.17%)
	Pungli River	6 (25.00%)	4 (16.67%)	3 (12.50%)	2 (8.33%)	4 (16.67%)	5 (20.83%)
	Jamuna River	5 (20.83%)	4 (16.67%)	2 (8.33%)	3 (12.50%)	3 (12.50%)	2 (8.33%)
	Total (21.35%)	28 (29.12%)	19 (19.80%)	12 (12.50%)	19 (19.80%)	24 (25.00%)	21 (21.90%)
Enrichment method	TMCSL	19 (79.16%)	14 (58.33%)	10 (41.67%)	17 (70.83%)	20 (83.33%)	16 (66.67%)
	Louhajang River	16 (66.67%)	13 (54.17%)	8 (33.33%)	11 (45.83%)	13 (54.17%)	15 (62.50%)
	Pungli River	15 (62.50%)	10 (41.67%)	5 (20.83%)	6 (25.00%)	10 (41.67%)	9 (37.50%)
	Jamuna River	12 (50.00%)	9 (37.50%)	4 (16.67%)	8 (33.33%)	7 (29.17%)	5 (20.83%)
	Total (47.20%)	62 (64.58%)	46 (48.00%)	27 (28.00%)	42 (43.75%)	50 (52.00%)	45 (46.88%)

The PEG method also showed higher recovery compared to the direct method (OR = 2.63, 95% CI: 1.87-3.70, p < 0.0001) (Table 2).

**Table 2:** Pairwise comparisons of bacteriophage recovery between three methods. Odds ratios (ORs), 95% confidence intervals (CIs), and p-values were calculated using two-sided Fisher’s exact test. A p-value < 0.05 was considered statistically significant. PEG, polyethylene glycol.

Method comparison	Odds ratio (OR)	95% CI	p-value
PEG vs. direct	2.63	1.87-3.70	< 0.0001
Enrichment vs. PEG	3.29	2.55-4.26	< 0.0001
Enrichment vs. direct	8.65	6.28-11.97	< 0.0001

Among the six bacteriophages, ETEC phage exhibited the highest recovery frequency across all methods, with average detection rates of 18.75%, 29.12%, and 64.58% using the direct, PEG, and enrichment methods, respectively. In contrast, *Shigella sonnei* phage showed the lowest recovery rates (3.13%, 12.50%, and 28.00%), indicating comparatively lower detection across sampled environments. Site-specific analysis revealed that the sewage-associated site TMCSL exhibited the highest overall phage recovery, with average rates of 18.01%, 31.95%, and 66.67% using the direct, PEG, and enrichment methods, respectively. In contrast, the Jamuna River consistently showed the lowest recovery (4.17%, 13.19%, and 31.25%).

Collectively, these findings demonstrate that the modified enrichment method substantially increases the likelihood of detecting bacteriophages across diverse host systems and environmental contexts, consistent with its higher observed recovery rates and strong effect sizes relative to PEG-mediated and direct approaches.

## Discussion

This comparative methodological study systematically evaluated three modified bacteriophage isolation strategies under standardized environmental conditions using environmental water samples collected from diverse aquatic sites. Across all sampling sites and host systems, the enrichment method demonstrated significantly higher phage recovery rates than both the direct and PEG-mediated methods. Statistical analysis confirmed significant differences in recovery efficiency among methods (χ² = 224.1, df = 2, p < 0.0001), with pairwise comparisons further demonstrating superior recovery by the enrichment method relative to both the PEG-mediated and direct approaches. These findings support the utility of host-based enrichment as a sensitive and robust strategy for isolating bacteriophages from complex environmental matrices.

The superior performance of the enrichment method is likely attributable to host-driven biological amplification, whereby low-abundance phages selectively replicate in the presence of susceptible bacterial hosts prior to detection. This amplification step enhances analytical sensitivity compared with the direct and PEG-mediated methods, which primarily depend on physical concentration of viral particles and may therefore be less effective when phage abundance is low. Similar observations have been reported previously, supporting the improved sensitivity of enrichment-based methods for environmental phage recovery [16,17].

Phage recovery varied substantially among host systems. Notably, phages targeting enterotoxigenic *Escherichia coli* (ETEC) demonstrated the highest recovery frequencies, suggesting broader environmental distribution or greater host prevalence in the sampled aquatic environments. In contrast, *Shigella sonnei* phages were detected less frequently, which may reflect lower environmental abundance, reduced persistence, or limited host availability.

The environmental context also influenced bacteriophage recovery. The sewage-associated site (TMCSL) consistently yielded higher recovery frequencies than riverine sampling locations, likely due to elevated bacterial densities, increased nutrient availability, and enhanced host-phage interactions in wastewater-associated environments. Previous studies have similarly identified sewage and wastewater systems as enriched reservoirs of bacteriophages because of their dense microbial communities and high organic load [18,19]. In contrast, reduced phage recovery in riverine systems, particularly the Jamuna River, may be explained by dilution effects, environmental heterogeneity, and lower concentrations of susceptible bacterial hosts.

Several limitations should be considered when interpreting these findings. First, plaque assessment was not performed under blinded conditions, which may introduce observational bias. Second, bacteriophage detection was based solely on culture-dependent plaque assays without molecular confirmation, such as quantitative polymerase chain reaction (qPCR), potentially limiting orthogonal validation of phage presence. Third, enrichment-based methods may preferentially amplify phages capable of infecting the selected host strains, thereby underrepresenting broader environmental phage diversity. Finally, environmental physicochemical parameters, including pH, temperature, turbidity, and bacterial load, were not systematically measured, limiting mechanistic interpretation of site-specific recovery differences.

Although the findings strongly support the application of the modified enrichment method for bacteriophage isolation from sewage-associated and aquatic environments, generalizability beyond the bacterial hosts and environmental settings examined in this study should be interpreted cautiously. Future studies integrating enrichment-based recovery with molecular or metagenomic approaches may further improve sensitivity while enabling broader characterization of environmental phage diversity.

## Conclusions

In this study, we demonstrate that a modified enrichment-based approach significantly improves bacteriophage recovery from environmental water samples compared with the conventional direct and PEG-mediated methods. This enhanced sensitivity is particularly valuable for detecting low-abundance phages, which may facilitate future applications in phage discovery, environmental surveillance, and the development of phage-based biocontrol or therapeutic strategies. Although the findings support enrichment as a robust and sensitive platform for bacteriophage isolation, further validation using molecular approaches and additional environmental settings is warranted to improve generalizability. Overall, the modified enrichment method provides a practical and reliable approach for environmental bacteriophage recovery and may support future large-scale phage screening efforts.
